# Ketamine promptly normalizes excess norepinephrine and enhances dopamine neuronal activity in Wistar Kyoto rats

**DOI:** 10.3389/fphar.2023.1276309

**Published:** 2023-10-31

**Authors:** Stephen Daniels, Mostafa El Mansari, Rami Hamoudeh, Pierre Blier

**Affiliations:** Institute of Mental Health Research, University of Ottawa, Ottawa, ON, Canada

**Keywords:** Wistar Kyoto, ketamine, serotonin, norepinephrine, dopamine, electrophysiology, depression

## Abstract

Ketamine acts primarily by blocking the N-methyl-D-aspartate (NMDA) receptor at the phencyclidine site. The rapid antidepressant properties of ketamine were demonstrated in the clinic and several behavioral models of depression in rodents. We hypothesized that the normalization of abnormal activity of monoamine neurons in Wistar Kyoto (WKY) rats contributes to the rapid antidepressant effects of ketamine. A single administration of ketamine (10 mg/kg, i. p) or saline was administered to anesthetized WKY rats before *in vivo* electrophysiological recordings of dorsal raphe nucleus (DRN) serotonin (5-HT), locus coeruleus (LC) norepinephrine (NE) and ventral tegmental area (VTA) dopamine (DA) neuronal activity. Pyramidal neurons from the medial prefrontal cortex (mPFC) were also recorded before and after a ketamine injection. In the VTA, ketamine elicited a significant increase in the population activity of DA neurons. This enhancement was consistent with findings in other depression-like models in which such a decreased population activity was observed. In the LC, ketamine normalized increased NE neuron burst activity found in WKY rats. In the DRN, ketamine did not significantly reverse 5-HT neuronal activity in WKY rats, which is dampened compared to Wistar rats. Ketamine did not significantly alter the neuronal activity of mPFC pyramidal neurons. These findings demonstrate that ketamine normalized NE neuronal activity and enhanced DA neuronal activity in WKY rats, which may contribute to its rapid antidepressant effect.

## Introduction

Clinical evidence demonstrates that a subanesthetic single dose of ketamine, a noncompetitive N-methyl-D-aspartate receptor (NMDAR) antagonist, induces a rapid antidepressant effect in treatment-resistant patients with major depressive disorder (MDD; McIntyre et al., 2021; Swainson et al., 2021). Preclinical studies have shown that ketamine increased the activation of α-amino-3-hydroxy-5-methyl-4-isoxazolepropionic acid receptor (AMPAR), brain-derived neurotrophic factor (BDNF) release and/or expression, activation of protein synthesis pathways (i.e., eEF2 kinase), increased expression of synaptic proteins (GluA1, PSD95, and synapsin), and increased synaptic number and function in the medial prefrontal cortex (mPFC; Duman et al., 2019; Hashimoto, 2019). These data indicate that the rapid antidepressant effect of ketamine is supported by increases in GluA1 and other synaptic proteins, in line with a reported enhancement in the formation and connectivity of new synapses. Importantly, studies also indicate that bidirectional interactions between glutamatergic pyramidal neurons in the PFC, and monoamine neurons in the dorsal raphe nucleus (DRN), locus coeruleus (LC), and ventral tegmental area (VTA) may contribute to the antidepressant response, including that of ketamine (Fukumoto et al., 2014; Nishitani et al., 2014; El Iskandrani et al., 2015; Fukumoto et al., 2016; Witkin et al., 2016; Hare et al., 2017; Lopez-Gil et al., 2019; Rincón-Cortés and Grace, 2020; Iro et al., 2021).

It was previously reported that reciprocal interactions between glutamate neurons in the PFC with serotonin (5-HT) neurons in the DRN play a fundamental role in the learned helplessness model of depression (Maier and Watkins, 2005). Indeed, the DRN gives rise to a majority of the 5-HT innervation of limbic and cortical brain regions, including the mPFC. On the other hand, the inactivation of mPFC at the time of stress exposure abolishes the influence of this structure in regulating stressor controllability (Amat et al., 2005), emphasizing the importance of this interaction in the deleterious effects of inescapable stress. Interestingly, microinjection of ketamine in the mPFC increases c-fos immunoreactivity in DRN 5-HT neurons (Fukumoto et al., 2016). In addition, the ketamine-induced reduction in the latency to feed in a novel environment was abolished by 5-HT depletion or by infusion of a 5- HT_1A_ receptor antagonist into the mPFC (Fukumoto et al., 2014; Fukumoto et al., 2016).

LC norepinephrine (NE) neurons are regulated by a stimulatory glutamatergic efferent emanating from mPFC pyramidal neurons, which in turn are controlled by NE innervation from LC. NE exerts an inverted U-shaped influence on PFC physiology and cognition. Accordingly, low levels of NE are found during fatigue, while phasic release takes place under alert and non-stress conditions, and high levels are observed during exposure to uncontrollable stress (Hains and Arnsten, 2008; Arnsten, 2009). The activation of postsynaptic α_2A_-adrenoceptor by NE inhibits irrelevant and distracting sensory processing by acting on glutamate pyramidal neurons of the PFC (Arnsten, 1998), which is thought to be beneficial in MDD. Interestingly, ketamine induces an increase in the NE level in mPFC (Lorrain et al., 2003a) and enhances climbing in the forced swim test (FST) in rodents (Lopez-Gil et al., 2019), interpreted as an antidepressant-like effect.

Dopamine (DA) and glutamate interact in a bidirectional manner to regulate reward processing. Anhedonia, which is a cardinal feature of depression (Yang et al., 2014; Daniels et al., 2021), is regulated by DA activity in the mesocorticolimbic pathway (Düzel et al., 2010). The glutamatergic tone has been shown to modulate mesolimbic functioning through a polysynaptic circuit projecting from the PFC onto midbrain DA neurons. Indeed, inhibition of VTA projections to the mPFC increased social avoidance in animals subjected to chronic social defeat stress (Chaudhury et al., 2013). Furthermore, acute ketamine administration rescued decreased sucrose consumption, a measure of anhedonia, in mice and rats exposed to chronic mild stress (Li et al., 2011) and also reversed depressive-like behavior in the FST (Rincón-Cortés and Grace, 2017). Finally, acute ketamine injection leads to enhancement in DA levels in the nucleus accumbens and the PFC (Moghaddam et al., 1997).

**FIGURE 1:**
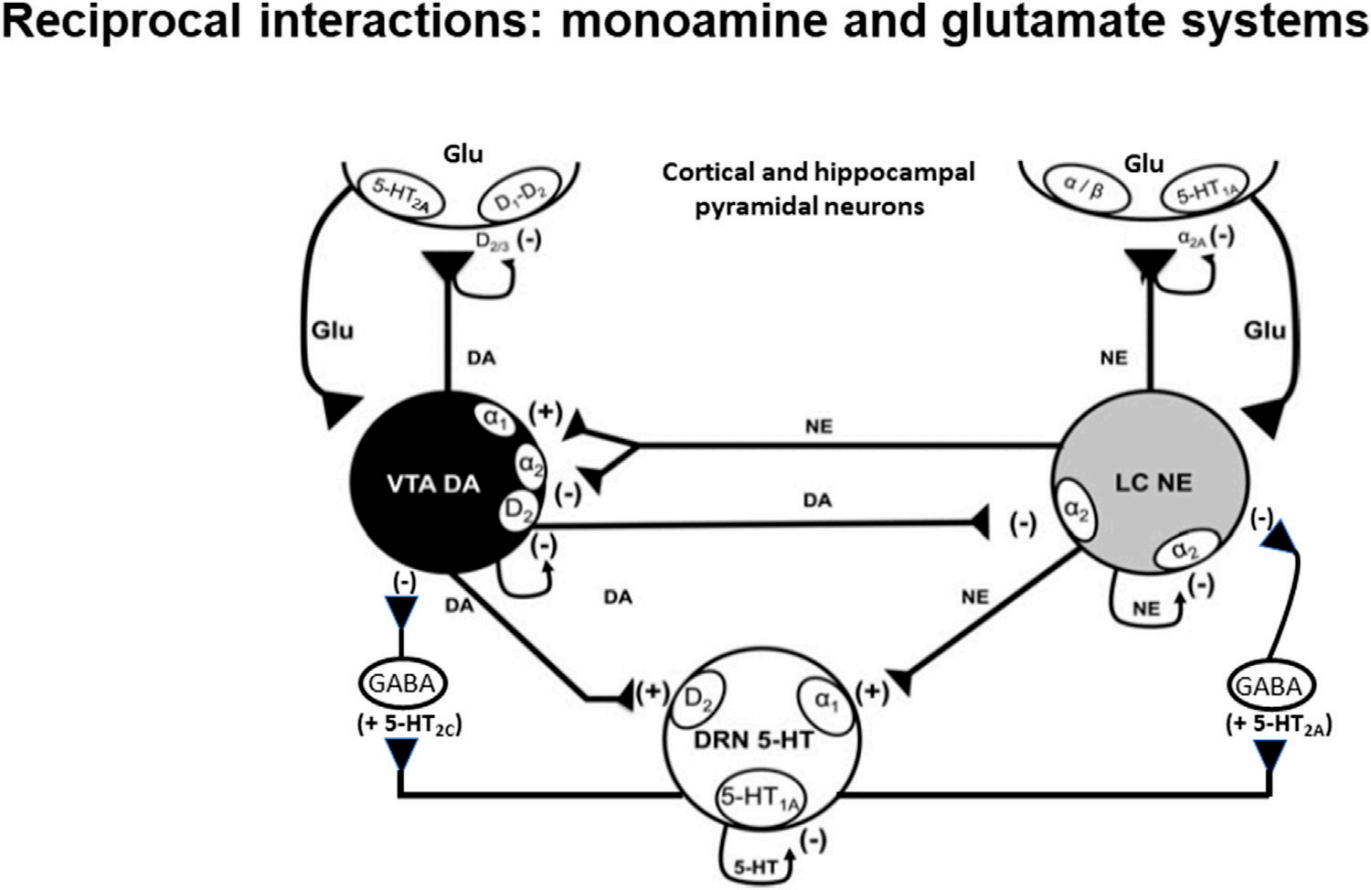
Schematic representation of the reciprocal interaction between glutamatergic pyramidal neurons and 5-HT, NE and DA neurons. It also depicts interplay between monoamine neurons (see Guiard et al., 2008). The nature of modulation of different neurotransmitters on classes of auto- and heteroreceptors is shown with (+) signs indicating a stimulatory effect and (−) signs indicating an inhibitory one.

Crosstalk between glutamate and monoamine pathways thus appears important in the antidepressant response and modulation of one system affect the others (Figure 1). Accordingly in the present study, the effects of a single administration of ketamine were examined on 5-HT, NE, DA, and mPFC neuronal activity in WKY, an animal model of depression.

## Materials and methods

### Animals

Adult male Wistar Kyoto (WKY) rats weighing approximately 280–350 g at the time of recording, were obtained from Charles River (St. Constant, Quebec, Canada). Animals were always kept under a constant temperature of 22°C in the facility and were housed in pairs under standard laboratory conditions (12:12-h light-dark cycle with *ad libitum* access to food and water). Rats were allowed 1 week to acclimatize to laboratory conditions. All animals were handled according to the guidelines of the Canadian Council on Animal Care (CCAC) and the local Animal Care Committee (University of Ottawa, Ottawa, Canada), which approved all protocols.

### Drug administration

Ketamine hydrochloride (10 mg/kg; ERFA Canada Inc.) was diluted in 0.9% saline solution and administered intraperitoneally (i.p.). Control rats received a single saline (0.9%) i. p. injection. All injections were given 30 min before electrophysiological recordings. Recordings were carried out starting 30 min after ketamine injection until 120 min after. This is in accordance with clinical findings (Zarate et al., 2006; Phillips et al., 2019) and animal studies including behavioral, molecular and electrophysiological reports showing important changes related to the antidepressant-like effects of ketamine can take place very rapidly (Li et al, 2010; Beurel et al, 2011; Lindholm et al., 2012; El Iskandrani et al., 2015; Lopez-Gil et al., 2019).

### *In Vivo* electrophysiological experiments

Rats were anesthetized using chloral hydrate (400 mg/kg i. p), and placed on a stereotaxic frame (David Kopf, CA, United States) with the skull positioned horizontally. Supplemental doses of chloral hydrate (100 mg/kg i. p) were given to maintain constant anesthesia to prevent any nociceptive response to the palpebral reflex or pinching of the hind paw (pedal withdrawal reflex). During electrophysiological recordings, rats were maintained at a constant body temperature of 37°C by a thermistor-controlled heating pad. A burr hole was drilled at the stereotaxic coordinates corresponding to the brain structure of interest. Electrode descents were carried out and neurons were identified and recorded for at least 2 min using the Spike2 program (Cambridge Electronic Design, Cambridge, United Kingdom).

Extracellular recordings of DRN 5-HT, LC NE, VTA DA, and mPFC pyramidal neurons were performed using single-barrel glass micropipettes (Stoelting, IL, United States) prepared using a pipette puller (Narishige, Japan) and filled with 2 M NaCl solution at an impedance range of 2–4 MΩ. Several electrode descents were carried out in each structure to record an optimal number of neurons (see detail below for VTA). The firing rate, number of neurons firing in bursts, percentage of spikes in bursts, and number of neurons per track were analyzed.

### Recording of DRN 5-HT neurons

5-HT neurons were recorded using a single-barrel glass micropipette positioned 0.9–1.2 mm anterior to lambda, on the midline, and lowered into the DRN, encountered at a depth of 4.5–5.5 mm from the surface of the brain. The following criteria were used to identify presumptive 5-HT neurons: a slow (0.5–2.5 Hz), regular firing rate, long duration (2–5 milliseconds [ms]), bi- or triphasic extracellular waveforms (Vandermaelen and Aghajanian, 1983).

### Recording of LC NE neurons

NE neurons were recorded using a single-barrel glass micropipette positioned at 1.1–1.2 mm posterior to lambda and 0.9–1.3 mm from the midline suture and at a depth of 4.5–6.0 mm from the surface of the brain. NE neurons were identified by their regular firing rate (0.5–5 Hz), a biphasic action potential of long duration (>2 ms), and a characteristic volley of spikes followed by a quiescent period in response to a nociceptive pinch of the contralateral hind paw (Marwaha and Aghajanian, 1982).

### Recording of VTA DA neurons

VTA DA neurons were recorded using a single-barrel glass micropipette positioned at 3.1–3.3 mm and 0.7–1.0 mm lateral to the midline and lowered to a depth of 6.5–9 mm from the surface of the brain. The number of spontaneously active DA neurons found per track was determined by recording multiple tracks in a grid of 6–9 tracks per rat. Presumed DA neurons were identified based on the following criteria (Ungless and Grace, 2012): 1) Regular or irregular single spiking pattern that may include burst firing with a rate between 2 and 10 Hz; 2) biphasic or triphasic waveforms, with an initial positive deflection (usually notched) followed by a prominent negative phase, with a duration >1.1 ms from start to trough of the waveform; 3) long-duration action potentials (2.5–4 ms), and 4) low-pitch sound when monitored by an audio amplifier.

### Recording of pyramidal neurons in the mPFC

Previous work has reported that pyramidal neurons in the mPFC are glutamatergic in nature (Santana et al., 2004). Putative mPFC pyramidal neurons were recorded by positioning a single-barrel glass micropipette according to the following coordinates (in mm from bregma): A-P, 3.2–3.4; M-L, 0.6–0.8; D-V, 2.5–5.5. Pyramidal neurons were identified according to the following electrophysiological properties: 1) A firing rate of 0.01–3 Hz, 2) a biphasic or triphasic action potential with irregular firing, and 3) a spike with a positive inflection duration greater than 0.36 ms and negative inflection duration greater than 1.08 ms to exclude any fast-spiking interneurons (Riga et al., 2017). In the acute experiments carried out in the mPFC, 9–23 pyramidal neurons were recorded in each rat to establish a mean baseline firing rate and a percentage of burst activity, then ketamine was injected. Subsequently, another 7–13 neurons were recorded in the same rat.

### Burst analyses

The firing activity of neurons was analyzed using spike-sorting software (www.github.com/nno/burstidator/releases). For burst activity, the start of a burst was indicated by the occurrence of 2 spikes with interspike intervals (ISI) < 0.08 s for NE and DA neurons and <0.01 s for 5-HT neurons. Burst termination was defined as an ISI >0.16 s for NE and DA (Grace and Bunney, 1983) and ISI >0.01 s for 5-HT neurons (Hajós and Sharp, 1996). For mPFC pyramidal neurons, burst determination was based on the following criteria: a series of 2 or more spikes, with ISI <0.045 s for the initiation and >0.045 s for termination of burst (Laviolette et al., 2005).

### Statistical analyses

All data are presented based on mean values ± standard error of the mean (SEM). Student’s t-tests were used to compare group means using two-tailed tests when the assumption of normality passed using the normality test Shapiro-Wilk. In cases where normality failed, the non-parametric Mann-Whitney test was utilized, which compared the median values of groups. In such cases, median values are indicated in the corresponding legend to figures. Statistical comparisons and graphing of data were conducted using SigmaPlot software 12.5 (Systat Software Inc., California, United States).

## Results

### Effect of ketamine on DRN 5-HT neuron activity in WKY rats

**FIGURE 2:**
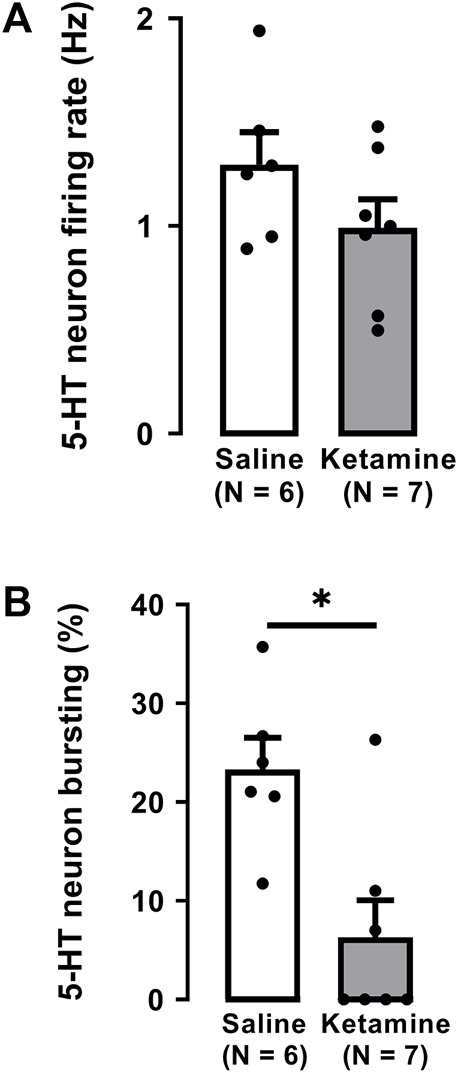
Effects of a single administration of ketamine (10 mg/kg, i. p.) on the firing rate **(A)** and burst activity **(B)** of 5-HT neurons in the DRN of WKY rats (number of rats = 7, number of neurons = 125) compared to WKY receiving saline (number of rats = 6, number of neurons = 139). Each dot represents an individual data point from one rat. The histograms show data as mean values ±SEM. Statistical significance is indicated where it applies, *p < 0.05.

As illustrated in Figure 2, a single administration of ketamine (10 mg/kg, i. p.) before electrophysiological recordings, did not significantly alter the firing rate of 5-HT neurons (saline: 1.3 ± 0.2 Hz; ketamine 1.0 ± 0.1 Hz; Two-tailed *t*-test, t [12] = 1.4, *p* = 0.2; Figure 2A).

A single administration of ketamine to WKY rats significantly decreased by 73% the number of neurons firing in burst mode when compared to saline administration (saline: 23% ± 3%; ketamine 6.3% ± 3.7%; Two-tailed *t*-test, t [11] = 3.4, *p* < 0.01; Figure 2B).

### Effect of ketamine on LC NE neuron activity in WKY rats

**FIGURE 3:**
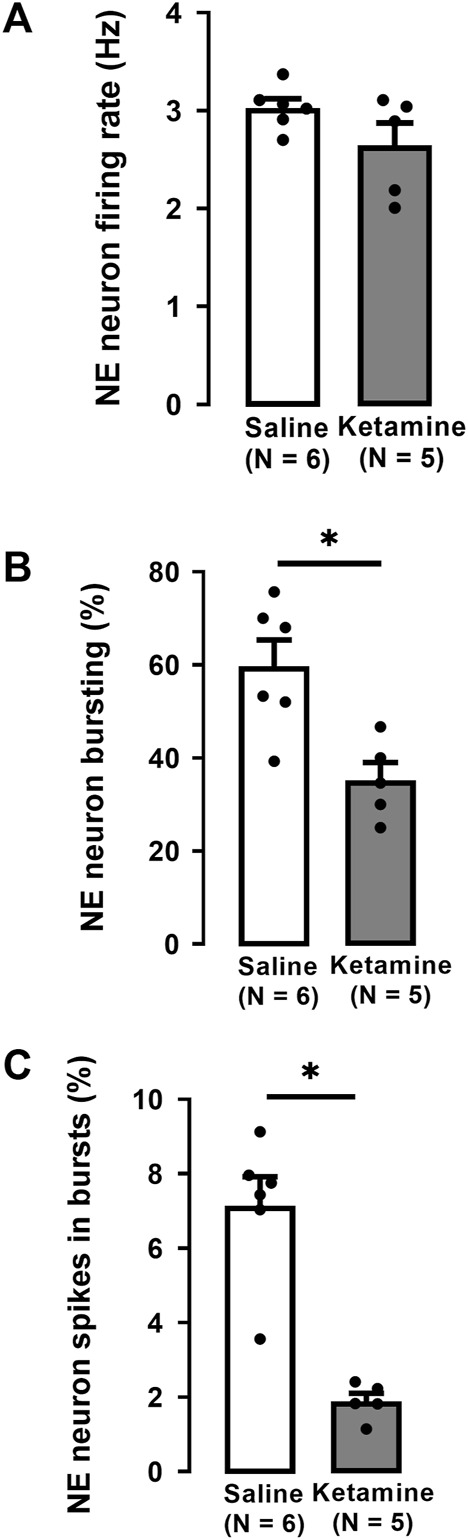
Effects of a single administration of ketamine (10 mg/kg, i. p.) on the firing rate **(A)**, percentage of neurons with bursts **(B)**, and the percentage of spikes in bursts **(C)** of NE neurons in the LC of WKY rats (number of rats = 5, number of neurons = 145) compared to WKY receiving saline (number of rats = 6, number of neurons = 175; median, saline: 7.6%, ketamine: 1.8%). Each dot represents an individual data point from one rat. The histograms show data as mean values ±SEM. Statistical significance is indicated where it applies, *p < 0.05.

There was no significant difference in the firing rate of NE neurons in WKY rats administered ketamine (3.0 ± 0.1 Hz) compared to WKY rats administered saline (2.7 ± 0.2 Hz; Two-tailed *t*-test, t [9] = 1.7, *p* = 0.1; Figure 3A).

A single administration of ketamine to WKY rats significantly decreased by 42% the number of NE neurons firing in burst mode compared to rats receiving saline (saline 60% ± 6%; ketamine 35% ± 4%; Two-tailed *t*-test, t [9] = 3.5, *p* < 0.01; Figure 3B). Similarly, the percentage of spikes in bursts was significantly decreased by 74% in WKY rats administered ketamine compared to saline (saline: 8% ± 0.7%; ketamine 2% ± 0.2%; Mann-Whitney *U* test, U = 0, *p* < 0.004; Figure 3C).

### Effect of ketamine on VTA DA neuron activity in WKY rats

**FIGURE 4:**
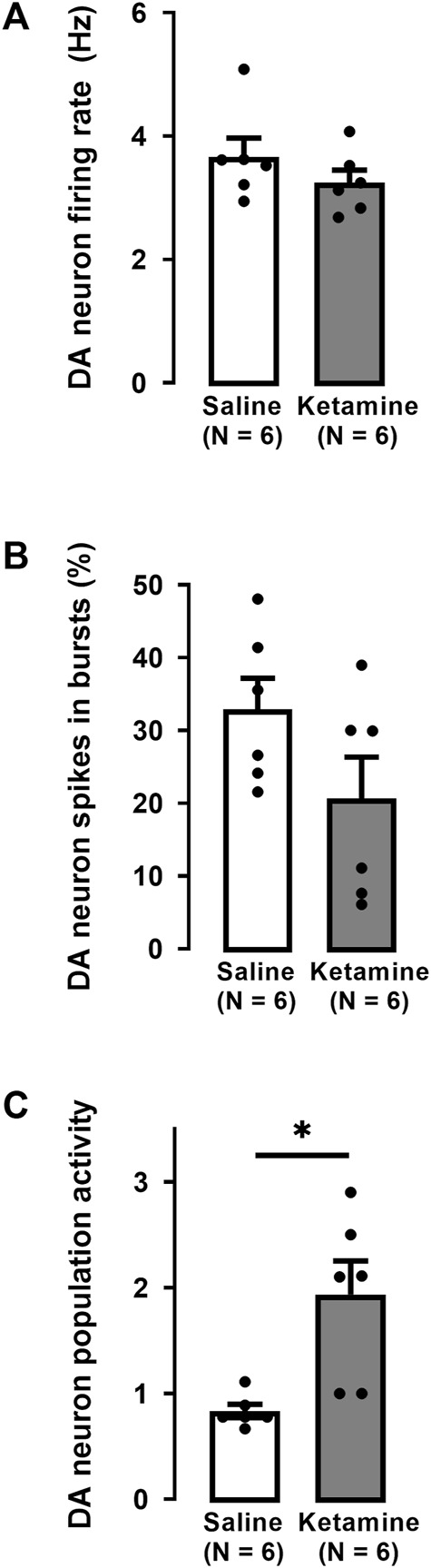
Effects of a single administration of ketamine (10 mg/kg, i. p.) on the firing rate **(A)**, percentage of spikes in bursts **(B)**, and population activity **(C)** of DA neurons in the VTA of WKY rats (number of rats = 6, number of neurons = 87) compared to WKY rats receiving saline (number of rats = 6, number of neurons = 45). Each dot represents an individual data point from one rat. The histograms show data as mean values ±SEM. Statistical significance is indicated where it applies, *p < 0.05.

In the VTA of WKY rats, a single administration of ketamine caused no significant effect on the firing activity of DA neurons (saline: 3.7 ± 0.3 Hz; ketamine 3.2 ± 0.2 Hz; Two-tailed *t*-test, t [10] = 1.1, *p* = 0.3; Figure 4A).

The percentage of spikes occurring in bursts was not significantly altered by ketamine compared to saline administration (saline: 28% ± 4%; ketamine 21% ± 6%; Two-tailed *t*-test, t [10] = 1.0, *p* = 0.4; Figure 4B). However, there was a two-fold enhancement of the number of spontaneously active DA neurons (population activity) after ketamine compared to saline administration (saline: 0.8 ± 0.06; ketamine: 1.9 ± 0.3; Two-tailed *t*-test, t [10] = −3.4, *p* < 0.01; Figure 4C).

### Effect of ketamine on mPFC pyramidal neuron activity in WKY rats

**FIGURE 5:**
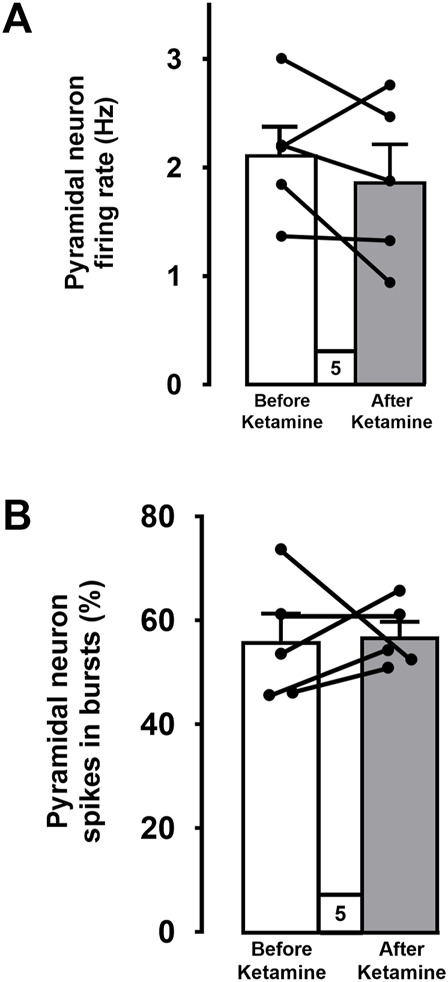
Mean firing rates **(A)** and percentages of spikes firing in bursts **(B)** of pooled data from 5 WKY rats before and after ketamine injections. Each dot represents an individual data point from one rat. Data points (before and after ketamine) from each rat are connected. The histograms show data as mean values ±SEM.

For each of the 5 WKY rats used in this series of experiments, a mean firing rate and a percentage of spikes in bursts of a population of mPFC pyramidal neurons was determined before and after the ketamine injection. When all data were pooled (Figure 5), there was no significant difference in the mean firing rate of neurons recorded before and after the ketamine injection (before: 2.1 ± 0.3 Hz, *n* = 53 versus 1.9 ± 0.3 Hz after ketamine, *n* = 60; 5 rats; Two-tailed *t*-test, t [4] = 1, *p* = 0.4; Figure 5A). The percentage of spikes in burst was also not altered following injection of ketamine (before: 56% ± 5% versus 56% ± 3% after ketamine; 5 rats; Two-tailed *t*-test, t [4] = −0.2, *p* = 0.9, Figure 5B).

## Discussion

Several lines of evidence show an involvement of 5-HT in the antidepressant-like effect of ketamine, including in the FST paradigm in which paracholoro-phenylanaline (PCPA) lowers the level of 5-HT and results in blunting of its effect on immobility in Sprague-Dawley (SD) rats (Gigliucci et al., 2013). In the current experiments, the mean firing rate of 5-HT neurons was similar in WKY rats that received ketamine compared to those that received saline. Considering the fact that 5-HT neuronal activity was decreased in WKY rats (Bruzos-Cidón et al., 2014; El Mansari et al., 2023), these results suggested that acute ketamine had no effect in restoring deficiency in the firing activity of DRN 5-HT neurons. Such a lack of effect of ketamine was not because 5-HT neurons activity was abnormally decreased in WKY rats, since even in naïve SD rats, where 5-HT neurons fire at control levels, acute injection of ketamine also did not alter the firing activity of 5-HT neurons (El Iskandrani et al., 2015). Such an absence of normalization of the firing of 5-HT neurons was also observed after administration of the selective 5-HT reuptake inhibitors (SSRIs) escitalopram and paroxetine, respectively, in heterozygous mice for 5-HT transporters (5-HTT^+/−^) and olfactory bulbectomized rats, that exhibit dampened firing activity of 5-HT neurons (Guiard et al., 2012; El Mansari et al., 2014). Nevertheless, it is possible to restore to normal levels, or even increase above baseline the firing activity of 5-HT neurons, using medications for MDD that act directly on monoamine receptors (Blier and El Mansari, 2013). For instance, the NE/DA releaser bupropion re-established normal firing in rats subjected to olfactory bulbectomy, which caused dampened firing activity (Dong and Blier, 2001; El Mansari et al., 2014). Normalization of 5-HT neuronal firing that is dampened following acute administration of an SSRI/selective norepinephrine reuptake inhibitor (SNRI) was, however, observed in SD rats that received an adjunct medication such as DA/5-HT modulators (Seager et al., 2004; Chernoloz et al., 2009; El Mansari et al., 2020). In addition, some antidepressant strategies for MDD such as vagus nerve stimulation, agomelatine, mirtazapine, bupropion, and pramipexole all enhance the firing activity of 5-HT neurons above their baseline in naïve SD rats (Blier and El Mansari, 2013). In these rats, the two latter drugs also increased 5-HT neuron burst activity (Chernoloz et al., 2009; Ghanbari et al., 2010), while this was significantly decreased by ketamine, here in WKY rats. This could not have been due to a direct action of ketamine on 5-HT neurons since the iontophoretic application of the NMDAR blocker, 2-APV, does not have any effect on their firing activity (Ferraro et al., 1997). It is conceivable that a dampening of the firing activity of 5-HT (including bursts) may have taken place indirectly through the activation of mPFC exerting an inhibition on 5-HT neurons (Celada et al., 2001). However, our present results showed that there was no increase in the firing activity of mPFC pyramidal neurons after ketamine injection and second, local blockade of NMDAR does not change the activity of these pyramidal neurons (Jodo et al., 2005). Nevertheless, the decrease in burst activity of 5-HT neurons by ketamine did not result in decreased levels of 5-HT in projection areas at least in SD rats. Microdialysis studies showed that 5-HT concentrations are enhanced in Wistar rat mPFC, but not the DRN. Although it was transient (1 h) after a systemic injection of 10 mg/kg of ketamine, 5-HT levels increased immediately after 25 mg/kg and lasted for at least 2 h (Lopez-Gil et al., 2012; Lopez-Gil et al., 2019). Indeed, non-competitive NMDAR antagonists selectively decrease GABA inhibition of pyramidal neurons resulting in their disinhibition and increase 5-HT levels in the mPFC (Adams and Moghaddam, 2001; Lopez-Gil et al., 2007). Altogether, these results suggest that the rapid antidepressant-like effect of ketamine is independent of the firing activity of 5-HT neurons, in line with results showing that the increase of c-fos-positive cells induced by NMDAR blockade was abolished by tetrodotoxin (TTX) in the mPFC, but not the DRN (Lopez-Gil et al., 2012).

Ketamine increases climbing in the FST, indicating the involvement of NE transmission in its antidepressant-like effect in Wistar rats (Lopez-Gil et al., 2019). In WKY rats, a previous electrophysiological study has also shown that the percentage of LC NE neurons displaying burst activity was significantly higher compared to Wistar rats by 41% (El Mansari et al., 2023). Interestingly, the present study showed in this same strain, that a single ketamine administration reduced by a similar proportion (42%) the number of NE neurons displaying burst activity and the percentage of spikes occurring in bursts as well. These observations taken together suggest that ketamine administration normalized maladaptive NE neuronal firing by decreasing the percentage of NE neurons exhibiting burst activity seen in WKY rats. This effect of ketamine could be mediated through direct blockade of NMDAR or via an increase in AMPAR activation following NMDAR blockade on GABA neurons (Miller et al., 2016; Widman and McMahon, 2018). It is unlikely that ketamine acts directly by blocking the NMDAR because it was shown that the selective NMDAR antagonist AP5 does not affect spontaneous NE firing activity (Charlety et al., 1993), although local and intracerebroventricular administration of the non-specific excitatory amino acid antagonist kynurenic acid decreases NE neurons burst activity (Tung et al., 1989; Saunier et al., 1993). An increase in AMPA throughput secondary to the blockade of NMDAR by ketamine is also not a likely explanation since AMPA does not affect the burst firing of NE neurons, albeit it enhances their mean firing activity (Olpe et al., 1989). The latter increase also cannot be due to the blockade of NMDARs in the mPFC because this would rather increase NE neuronal discharge (Jodo et al., 1998). Therefore, how ketamine decreases burst activity in WKY rats remains to be elucidated.

In SD rats, a single ketamine injection induced an increase in the firing and burst activity of NE neurons (El Iskandrani et al., 2015). The opposite effects of ketamine observed in WKY rats herein, versus SD rats, may be attributable to a difference in basal NE transmission between the two strains. Such differences include lower levels of NE and desensitized α_2_-adrenoceptors on the cell body in the LC and terminals in the hippocampus of WKY rats (Scholl et al., 2010; Jedema et al., 2008; Bruzos-Cidón et al., 2014; El Mansari et al., 2023). Whether an increase of NE neuronal activity as in WKY rats is a maladaptation or rather constitutes a beneficial adaptation induced by drugs with antidepressant-like action in SD rats remains to be elucidated. Nevertheless, similar increased phasic NE transmission is also manifested in behavioral and neuropsychological functions of patients experiencing anxiety, particularly those with post-traumatic stress syndrome (Hains and Arnsten, 2008; Arnsten, 2009). These manifestations seem to be mimicked by an enhanced phasic burst activity as in WKY rats (El Mansari et al., 2023; Bruzos-Cidón et al., 2014). On the other hand, an enhanced NE transmission through an increase in firing and/or burst activity, as well as tonic activation of α_2_-adrenoceptors, was also reported using several antidepressant strategies modulating 5-HT, NE, and DA systems in naïve SD rats (Blier and El Mansari, 2013). It was hypothesized that increasing tonic NE function within their basal firing range consequently dampens excessive phasic firing through activation of the inhibitory α_2_-adrenergic autoreceptors (see Montoya et al., 2016). The present results suggest that ketamine decreased the burst activity of NE neurons while their tonic firing remained at basal levels in WKY rats. In SD naïve rats, however, the firing activity of NE neurons was dampened following acute and long-term administration of medications acting directly on the NE system, including the NE reuptake inhibitors reboxetine and desipramine (Lacroix et al., 1991; Szabo and Blier, 2001a). It has been postulated that the antidepressant response induced by these medications is rather based on a tonic increase in NE in projection areas, such as the frontal cortex and hippocampus, due to a desensitization of terminal α_2_-adrenoceptors (Szabo and Blier, 2001b; Invernizzi et al., 2001). Regardless of NE neuronal firing pattern, microdialysis studies in SD rats have shown that acute ketamine (12.5–25 mg/kg) increased, for at least 2 h, NE levels in the mPFC and the ventral hippocampus (Lorrain et al., 2003a; Lopez-Gil et al., 2019). This is congruent with an increase in climbing in the FST (Lopez-Gil et al., 2019) and thus indicates a net augmentation in NE transmission (Detke et al., 1995). Altogether, these data imply that ketamine normalized phasic NE neuronal firing in WKY rats, and increased tonic NE transmission found in SD rats, which may underlie at least in part the rapid antidepressant action of ketamine. A similar augmentation in NE transmission is also achieved with standard antidepressant treatments, but only after chronic administration (El Mansari et al., 2020).

Previous studies in SD rats documented increased population activity of DA neurons by acute administration of ketamine, while firing and burst activity was unchanged (El Iskandrani et al., 2015; Witkin et al., 2016). A similar increase was also observed herein, utilizing the same regimen in WKY rats, where the firing properties of DA neurons were previously shown not to be different from those of Wistar rats (El Mansari et al., 2023). Since 5-HT and NE neurons exert an inhibitory effect on DA neurons activity (Figure 1; Guiard et al., 2008), it is possible that decreased neuronal activity of 5-HT and NE in WKY rats that received ketamine resulted in an increase in DA neurons activity, albeit the effect on population activity remains to be elucidated. Population activity of DA neurons, however, is regulated by the ventral subiculum where a local infusion of NMDA elicits an increase in this parameter (Floresco et al., 2003). Hence, it is unlikely that the increase in DA neuron population activity observed herein is due to the direct blockade of NMDAR *per se* because this effect induces rather a decrease in the number of DA neurons recorded per track (Floresco et al., 2003). It is possible, however, that the observed ketamine-induced increase is instead due to the promotion of AMPAR-mediated glutamatergic neurotransmission, as previously hypothesized (Miller et al., 2016; Widman and McMahon, 2018). Indeed, previous results had shown an increase in the firing of DA neurons after the local application of AMPA in the VTA, although the effect on burst was not assessed (Tong et al., 1996; Zhang et al., 1997). In learned helpless rats, another model of depression, showing dampened population activity of VTA DA neurons, ketamine rescued this deficit and also increased the basal number of spontaneously active DA neurons (Belujon and Grace, 2014). A similar restoration of dampened population activity of DA neurons was also reported in male and female rats subjected to chronic mild stress (Rincón-Cortés and Grace, 2017). Altogether, these results show that ketamine can increase DA neuronal activity, particularly population activity, which produces a significant enhancement in DA concentration in the nucleus accumbens (Floresco et al., 2003; Lisman and Grace, 2005), and modulates lower reward expectation (i.e., anhedonia; Markovic et al., 2021). Consistent with this interpretation, acute ketamine administration (10–25 mg/kg) increased DA levels, for at least 1 h, in the mPFC, although in naïve SD rats (Lorrain et al., 2003b; Lindefors et al.,1997; Moghaddam et al., 1997). As with ketamine, classical medications indicated for MDD administered alone or in combination with DA/5-HT modulators (i.e., aripiprazole, cariprazine) either increased above basal level or normalized dampened DA neurons firing/bursts/population activity, only following repeated but not acute regimens (Chernoloz et al., 2009; El Mansari et al., 2020). Thus, it is probable that an increase in DA transmission contributes significantly to the rapid antidepressant effect of ketamine.

Previous work has demonstrated that local injection of ketamine into the mPFC is sufficient to reproduce rapid antidepressant-like action in rats (Fuchikami et al., 2015). Furthermore, optogenetic stimulation of the infralimbic part of the PFC induces a rapid antidepressant-like effect, as measured in behavior and synaptogenesis (Fuchikami et al., 2015), indicating an important contribution of mPFC neuronal activity. Indeed, disinhibited activity of pyramidal neurons following a selective reduction of GABAergic inhibition is considered a possible mechanism of action of ketamine (Miller et al., 2016; Widman and McMahon, 2018). This would be consistent with an increased efflux of glutamate within the mPFC by ketamine (Moghaddam et al., 1997; Lopez-Gil et al., 2007), resulting in an enhancement of the firing rate of these glutamatergic neurons. Although the local infusion of ketamine enhanced c-fos immunolabeling in the infralimbic part of the PFC, the present study showed no change in the mean firing and burst activity of mPFC pyramidal neurons after acute ketamine administration. Our results are also inconsistent with a previous study showing that cumulative injections of ketamine (1.25–20 mg/kg, i. v.) inhibited the firing activity of pyramidal neurons in the PFC (Shen et al., 2018). Such a discrepancy may stem from the fact the latter study assessed the effect of ketamine injection on single neurons in each rat, while the current study assessed the mean firing of a population of neurons before and after i. p. ketamine, which may reflect more the average change in such a neuronal population. Moreover, in the majority of a population of pyramidal neurons, their mean firing was enhanced following the blockade of NMDARs using MK-801, another NMDAR antagonist (Homayoun and Moghaddam, 2007). PCP administered systemically resulted in half of PFC pyramidal neurons increasing their firing activity, while the rest either decreased or showed no variation (Kargieman et al., 2007). The current approach sampling the firing activity of PFC pyramidal neurons before and after ketamine injection did not reveal the crucial role of this brain structure in the antidepressant-like effect of ketamine. However, the action of ketamine and especially regarding a subpopulation of mPFC pyramidal neurons projecting to monoamines neurons remains to be further elucidated.

As illustrated in Table 1, the present study showed that in the DRN, the acute injection of ketamine did not change the firing activity of 5-HT neurons in WKY rats, where activity is below normal, although previously it did increase 5-HT levels in the mPFC and the hippocampus. In the LC, acute ketamine administration reversed abnormally higher burst activity of NE neurons in WKY rats. In the VTA, ketamine enhanced population activity, which is similar to the effects of several medications used to treat MDD. Ketamine promptly normalized or enhanced monoamine neurotransmissions, indicating that such alterations can contribute, at least in part, to its rapid antidepressant-like effects.

**TABLE 1:** Summary of effects of a single administration of ketamine (10 mg/kg) in WKY rats on the firing and burst activity (% spikes in bursts) of DRN 5-HT, LC NE and mPFC glutamatergic (Glu) pyramidal neurons. For the VTA, DA neurons population activity was also included. The upward and downward arrows represent, respectively, a significant increase or decrease compared to saline administered WKY rats. The circle with a line through represents no significant change.

	DRN 5-HT neurons		LC NE neurons		VTA DA neurons			mPFC Glu neurons	
	Firing rate	Bursts	Firing rate	Bursts	Firing rate	Bursts	Population activity	Firing rate	Bursts
Acute Ketamine	∅	↓	∅	↓	∅	∅	↑	∅	∅

## Study limitations

In the current studies, experiments in both vehicle- and ketamine-treated rats were carried out under chloral hydrate anesthesia, to ensure a similar basal condition in both groups. The assumption then is that any difference between these two groups would be attributable to effects induced by ketamine. Furthermore, several lines of evidence show that ketamine effects are more likely in opposite direction to chloral hydrate. On the one hand, chloral hydrate through its metabolite trichloroethanol enhances GABA_A_-mediated transmission, which is responsible for its anesthetic effects (Lovinger et al., 1993; Krasowski and Harrison, 2000). In addition, its potency to enhance this GABA effect appears to be greater than to depress excitatory transmission (Lovinger et al., 1993). Moreover, chloral hydrate inhibits the AMPA response by 50% (Carlà and Moroni, 1992; Peoples and Weight, 1998; Fischer et al., 2000). On the other hand, ketamine induces a disinhibition of GABA neurons through a blockade of NMDA receptors, which results in an increase in AMPA throughput (Li et al., 2010; Miller et al., 2016; Widman and McMahon, 2018). Therefore, it is possible that effects obtained after ketamine administration herein are even more likely to be under-estimated because of the opposing action of chloral hydrate on the impact of ketamine.

Although these findings are limited to male rats, clinical studies have shown no difference in the effects of a single ketamine infusion in women and men with treatment-resistant depression, even when menopause status is considered (Freeman et al., 2019; Phillips et al., 2019).

## Data Availability

The raw data supporting the conclusion of this article will be made available by the authors, without undue reservation.
