# Supplementary figures and images for: Ketamine promptly normalizes excess norepinephrine and enhances dopamine neuronal activity in Wistar Kyoto rats
Source: Front Pharmacol. 2023 Oct 31;14:1276309. doi: 10.3389/fphar.2023.1276309 (PMC10644068; doi:10.3389/fphar.2023.1276309)

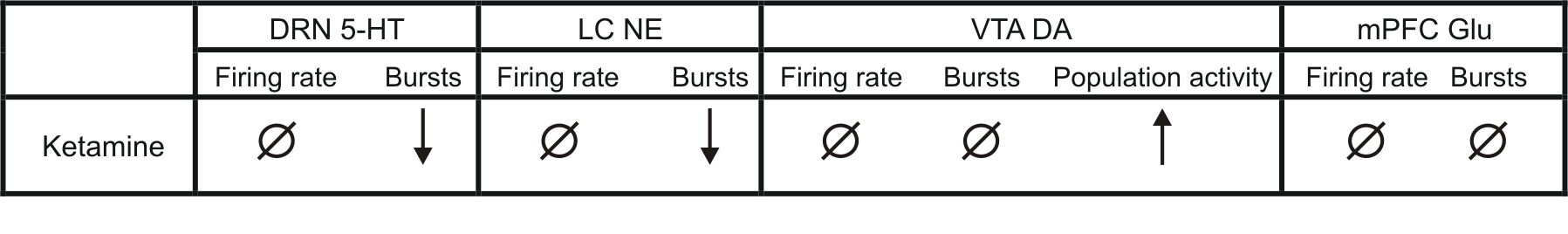

Supplement: Supplementary file 1 [file Image1.TIF]
